# Supplementary material for: Importance of dxy orbital and electron correlation in iron-based superconductors revealed by phase diagram for 1111-system
Source: Sci Rep. 2021 May 11;11:10006. doi: 10.1038/s41598-021-89231-2 (PMC8113352; doi:10.1038/s41598-021-89231-2)
Supplement: Supplementary file 1 — Supplementary Information. [file 41598_2021_89231_MOESM1_ESM.pdf]

Supplementary Information : Importance of d_{xy} orbital and electron correlation in iron-based superconductors revealed by phase diagram for 1111-system

Tsuyoshi Kawashima[1], Shigeki Miyasaka[1,*], Hirokazu Tsuji[1], Takahiro Yamamoto[1], Masahiro Uekubo[1], Akira Takemori[1], Kwing To Lai[1,2], and Setsuko Tajima[1]

[1]Department of Physics, Osaka University, Toyonaka, Osaka, 560-0043, Japan
[2]Department of Physics, The Chinese University of Hong Kong, Shatin, Hong Kong
[*]miyasaka@phys.sci.osaka-u.ac.jp

Structural Parameters

All the polycrystalline samples of RFeAs$_{1-x}$(P/Sb)$_x$O$_{1-y}$(F/H)$_y$ (R = La and Nd) were characterized by synchrotron X-ray diffraction. Figure S1 shows the x-dependence of the lattice constants a and c, the pnictogen (Pn) height from the Fe-layer (h_{Pn}), the Pn-Fe-Pn bond angle (α), and the Fe-Pn bond distance ($d_{\mathrm{Fe}-Pn}$) in RFeAs$_{1-x}$(P/Sb)$_x$O$_{1-y}$(F/H)$_y$ (R = La and Nd). The x-dependence of the lattice constants for LaFeAs$_{1-x}$P$_x$O and NdFeAs$_{1-x}$P$_x$O$_{0.95}$F$_{0.05}$ is also plotted in Figs. S1(a) and (b) for reference. The crystallographic parameters for other samples with different y-values have been shown in Fig. 1 and in our previous report[1].

In all samples of RFeAs$_{1-x}$(P/Sb)$_x$O$_{1-y}$(F/H)$_y$, the lattice constants show linear x-dependence, indicating that P or Sb was successfully substituted for As. In addition, the structural parameters have been systematically changed by F- and H-doping. The local structural parameters α and $d_{\mathrm{Fe}-Pn}$ were used when the contour plot of superconducting transition temperature T_c was generated in Fig. 6.

Temperature Dependence of Resistivity

Figures S2 and S3 present the temperature (T-) dependence of resistivity for NdFeAs$_{1-x}$P$_x$O$_{1-y}$(F/H)$_y$ and LaFeAs$_{1-x}$Sb$_x$O$_{1-y}$(F/H)$_y$, respectively. The values of T_c were determined from T for zero resistivity. The power n in $\rho(T) = \rho_0 + AT^n$ was estimated by fitting $\rho(T)$. These T_c and n values were shown in Figs. 4, 5 and 6. The T-dependent resistivities for other compositions were shown in Fig. 2 and our previous reports[1–3].

References

1. Miyasaka, S. *et al.* Three superconducting phases with different categories of pairing in hole- and electron-doped LaFeAs$_{1-x}$P$_x$O. *Phys. Rev. B* **95**, 214515 (2017).
2. Miyasaka, S. *et al.* Two Fermi Surface States and Two T_c-Rising Mechanisms Revealed by Transport Properties in RFeP$_{1-x}$As$_x$O$_{0.9}$F$_{0.1}$ (R = La, Pr, and Nd). *J. Phys. Soc. Jpn.* **82**, 124706 (2013).
3. Lai, K. T. *et al.* Evolution of the phase diagram of LaFeP$_{1-x}$As$_x$O$_{1-y}$F$_y$ ($y = 0 - 0.1$). *Phys. Rev. B* **90**, 064504 (2014).

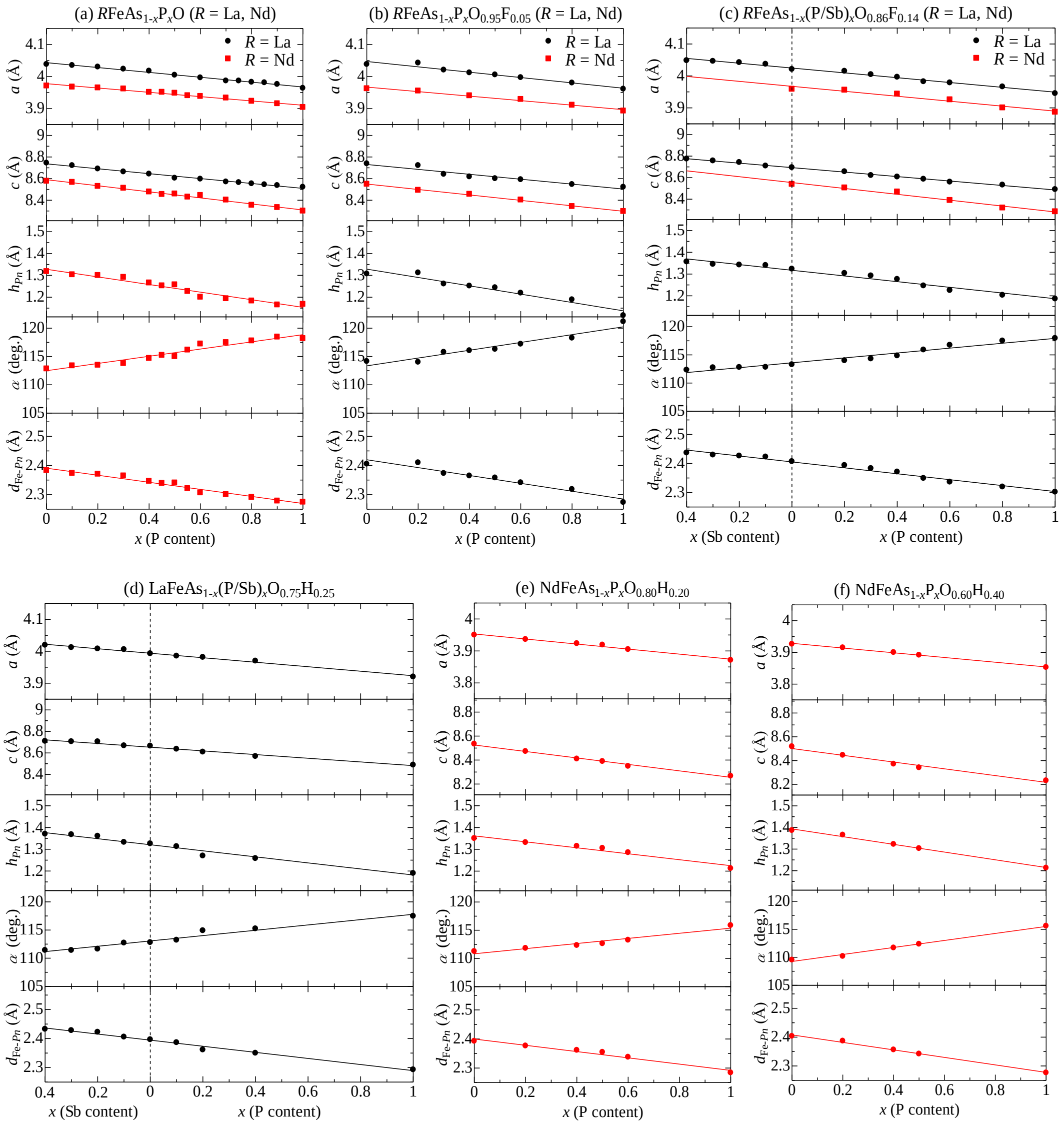

Figure S1. x-dependence of structural parameters: lattice constants a and c, pnictogen height from the Fe plane h_{Pn}, Pn-Fe-Pn bond angle α, and Fe-Pn bond length $d_{\mathrm{Fe}-Pn}$, in (a) $R\mathrm{FeAs}_{1-x}\mathrm{P}_x\mathrm{O}$ (R = La and Nd), (b) $R\mathrm{FeAs}_{1-x}\mathrm{P}_x\mathrm{O}_{0.95}\mathrm{F}_{0.05}$, (c) $R\mathrm{FeAs}_{1-x}(\mathrm{P/Sb})_x\mathrm{O}_{0.86}\mathrm{F}_{0.14}$, (d) $\mathrm{LaFeAs}_{1-x}(\mathrm{P/Sb})_x\mathrm{O}_{0.75}\mathrm{H}_{0.25}$, (e) $\mathrm{NdFeAs}_{1-x}\mathrm{P}_x\mathrm{O}_{0.80}\mathrm{H}_{0.20}$, and (f) $\mathrm{NdFeAs}_{1-x}\mathrm{P}_x\mathrm{O}_{0.60}\mathrm{H}_{0.40}$.

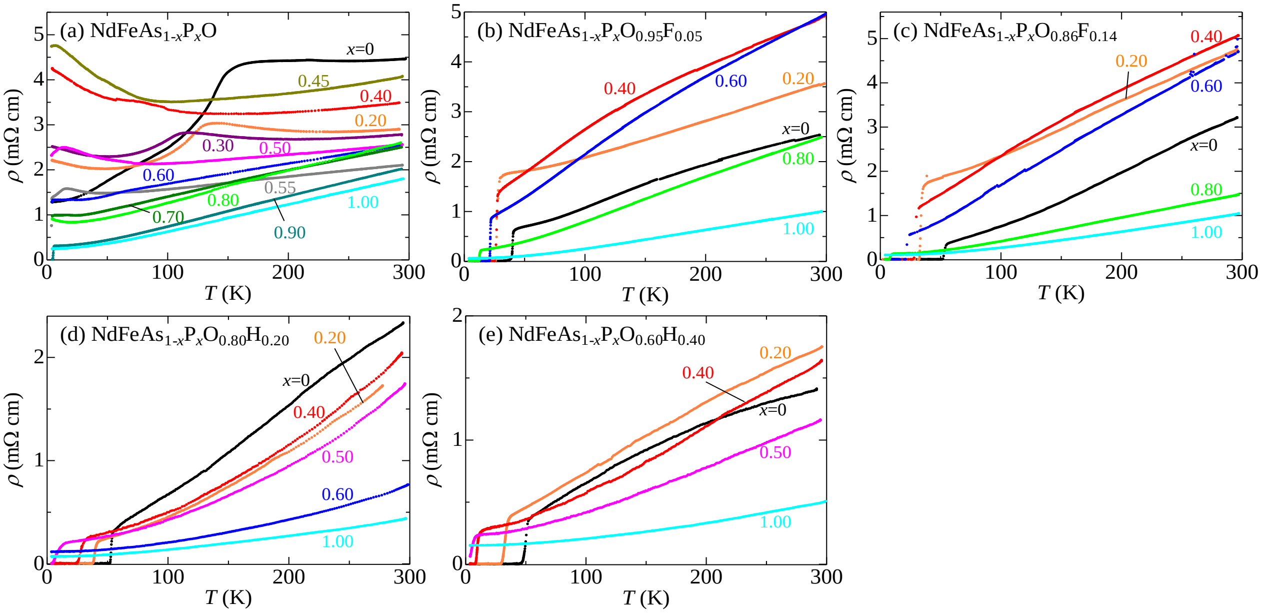

Figure S2. Temperature (T-) dependence of resistivity ρ for (a) $NdFeAs_{1-x}P_xO$, (b) $NdFeAs_{1-x}P_xO_{0.95}F_{0.05}$, (c) $NdFeAs_{1-x}P_xO_{0.86}F_{0.14}$, (d) $NdFeAs_{1-x}P_xO_{0.80}H_{0.20}$, and (e) $NdFeAs_{1-x}P_xO_{0.60}H_{0.40}$ for various values of x.

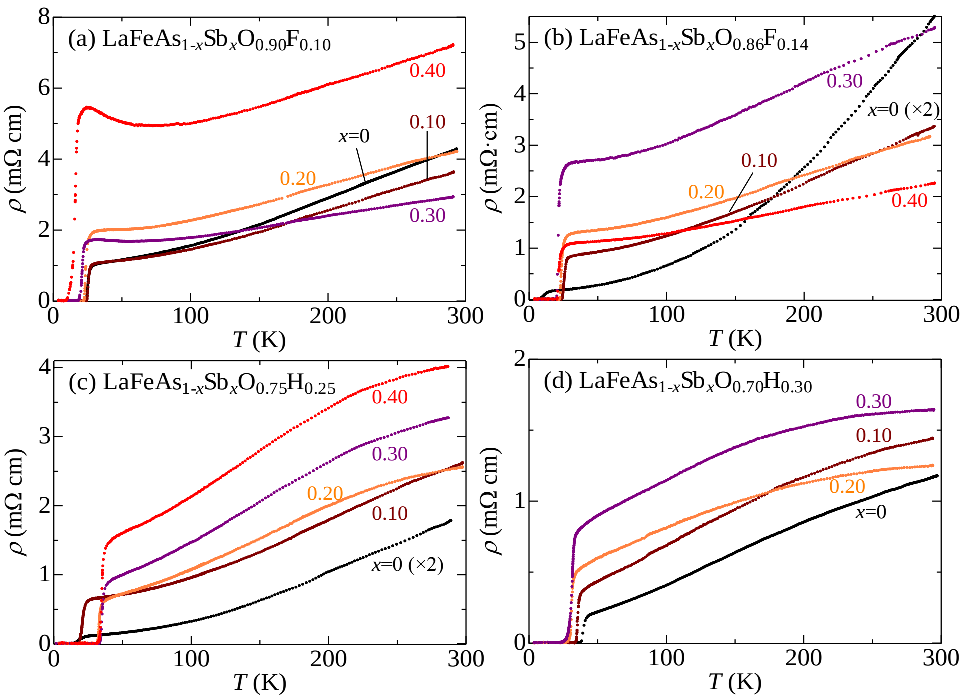

Figure S3. Temperature (T-) dependence of resistivity ρ for (a) $LaFeAs_{1-x}Sb_xO_{0.90}F_{0.10}$, (b) $LaFeAs_{1-x}Sb_xO_{0.86}F_{0.14}$, (c) $LaFeAs_{1-x}Sb_xO_{0.75}H_{0.25}$, and (d) $LaFeAs_{1-x}Sb_xO_{0.70}H_{0.30}$, with various values of x.
